# Bridging the Science–Policy Divide

**DOI:** 10.1371/journal.pbio.0020027

**Published:** 2004-02-17

**Authors:** Walter V Reid

## Abstract

The Millennium Ecosystem Assessment focuses on the benefits people obtain from ecosystems and aims to improve ecosystem management and contribute to human well-being and poverty alleviation

Nobody questions the importance of good scientific information for sound environmental decision-making. But designing mechanisms to link scientific research to the decision-making process is no easy matter. Research and decision-making often seem to operate in different worlds. Policy-makers' needs for applied findings and best judgment typically clash with scientists' pursuit of basic research and statistical significance.

Despite this challenge, as needs for scientific input into decision-making arise, a number of institutions have been established to help bridge the science–policy divide. Regulatory agencies like the United States' Food and Drug Administration have met this need in areas of public health, for example, and Environmental Impact Assessment procedures have helped to introduce better science into project-level decisions. At the scale of global environmental challenges, highly regarded mechanisms have been established such as the Scientific Assessment of Ozone Depletion, which has guided decisions by governments, the private sector, and nongovernmental organizations (NGOs), and the Intergovernmental Panel on Climate Change, which has become the authoritative source of policy-relevant information on climate science.

But a significant gap remains in the landscape of institutions designed to link science with policy-making: no mechanism has existed to provide decision-makers with authoritative information on the causes and consequences of changes in the planet's ecosystems and on the options for response.

Human well-being and progress toward sustainable development are vitally dependent upon improving the management of Earth's ecosystems to ensure their conservation and sustainable use. The benefits that human beings extract from nature are the foundation of all economies and the basis of major industries, are sources of knowledge, and are central to many cultures. While demands for ecosystem services such as food and clean water are growing, human actions are at the same time diminishing the capability of many ecosystems to meet these demands. And while many of the changes to ecosystems, such as increased agricultural production, have greatly enhanced human well-being, many others have not. World fisheries are now declining due to overfishing, for instance, and some 40% of agricultural land has been degraded in the past half-century. Other human-induced impacts on ecosystems include alteration of the nitrogen, phosphorous, sulfur, and carbon cycles, causing acid rain, algal blooms, and fish kills in rivers and coastal waters, along with contributions to climate change.

*The benefits that human beings extract from nature are the foundation of all economies and the basis of major industries, are sources of knowledge, and are central to many cultures.*

Recognizing the growing scale of these problems, United Nations Secretary General Kofi Annan, in his 2000 Millennium Report to the General Assembly, called for a Millennium Assessment of Global Ecosystems to provide definitive information on the consequences of ecosystem change for human well-being. With further authorization received through three international conventions (on Biological Diversity, Desertification, and Wetlands) and with financial support from the Global Environment Facility, the United Nations Foundation, the David and Lucile Packard Foundation, and the World Bank, the Millennium Ecosystem Assessment (MA) (www.millenniumassessment.org) was launched one year later, in 2001. More than 700 authors from 80 countries are now involved in the expert working groups preparing the global assessment; 100 experts will serve on the Editorial Review Board; more than 1,000 experts will be asked to review the materials, and hundreds more are undertaking subglobal assessments as part of the MA.

The MA focuses on ecosystem services (the benefits people obtain from ecosystems), how changes in ecosystem services have affected human well-being, how ecosystem changes may affect people in future decades, and response options that might be adopted at local, national, or global scales to improve ecosystem management and thereby contribute to human well-being and poverty alleviation (Figure 1).

**Figure 1:**
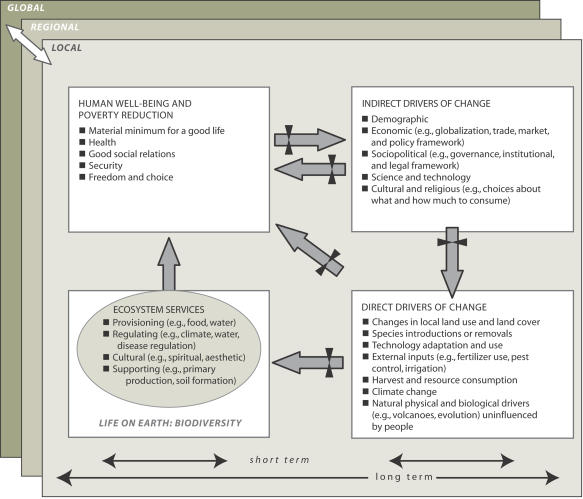
MA Conceptual Framework The MA examines both indirect and direct drivers (both human-caused and natural) of change in ecosystems, how those changes affect ecosystem services, how those changes, in turn, influence human well-being and poverty reduction, and opportunities for interventions that can ensure ecosystem conservation and enhance human well-being. The assessment must take into consideration the multiple time and spatial scales over which these interactions take place. (Schematic is used by permission from the Millennium Ecosystem Assessment [2003] and published under the terms of the Creative Commons Attribution License.)

The first report—*Ecosystems and Human Well-Being: A Framework for Assessment*—was published in 2003 and describes the approach and methods used in the MA. The four main assessment volumes—Conditions and Trends, Scenarios, Response Options, and Subglobal Assessments—began the first of two rounds of peer review in January 2004, and the final assessment reports will be published in early 2005.

Unlike previous global scientific assessments, the MA is a “multiscale” assessment. Assessments at subglobal scales are needed because ecosystems are highly differentiated in space and time and because sound management requires careful local planning and action. Local assessments alone are insufficient, however, because some processes are global and because local goods, services, matter, and energy are often transferred across regions. The MA subglobal assessments will directly meet needs of decision-makers at the scale at which they are undertaken, strengthen the global findings with on-the-ground reality, and reinforce the local findings with global perspectives, data, and models.

In Southern Africa, for example, a series of community-level assessments are being conducted using the MA conceptual framework. The findings from these assessments inform, and are informed by, assessments underway in the Gariep and Zambezi river basins. These local and river basin assessments, in turn, are linked to a regional assessment encompassing the countries in the Southern African Development Community. Other subglobal assessments are now underway in such regions as São Paulo, Brazil; coastal British Columbia, Canada; the Caribbean Sea; western China; Colombia; the Sinai Peninsula, Egypt; several regions within India; Indonesia; the Laguna Lake Basin, the Philippines; Portugal; and Sweden.

The ultimate impact of the MA will depend on its credibility, legitimacy, and utility. To ensure its scientific credibility, the assessment involves leading scientists from around the world and has established an independent peer-review process. To ensure the political legitimacy, all of the stakeholders—governments, the private sector, and NGOs—have a role in governing the process, and governments themselves have approved the process through decisions in international conventions. And, to ensure its utility, ongoing interactions with stakeholders are designed to ensure a focus on their questions and issues.

The scientific information now available concerning ecosystems and human development holds the promise of significantly improving the choices that the public and decision-makers take concerning the environment. But for that promise to be fulfilled, a bridge needs to be built between the research community holding this information and the decision-makers seeking it. The MA is an attempt to establish that bridge.

## Abbreviations

MA: Millennium Ecosystem Assessment
NGO: nongovernmental organization
